# A Pixel Circuit for Compensating Electrical Characteristics Variation and OLED Degradation

**DOI:** 10.3390/mi14040857

**Published:** 2023-04-15

**Authors:** Ning Wei, Hongzhen Chu, Bo Yu, Huicheng Zhao, Yuehua Li, Xinlin Wang, Hongyu He

**Affiliations:** 1 School of Electrical Engineering, University of South China, Hengyang 421001, China; 2 School of Electronics and Information, Yangtze University, Jingzhou 434023, China

**Keywords:** pixel circuit, voltage programming, threshold voltage variation, mobility variation, *OLED* degradation

## Abstract

In recent years, the active-matrix organic light-emitting diode (AMOLED) displays have been greatly required. A voltage compensation pixel circuit based on an amorphous indium gallium zinc oxide thin-film transistor is presented for AMOLED displays. The circuit is composed of five transistors–two capacitors (5*T*2C) in combination with an *OLED*. In the circuit, the threshold voltages of both the transistor and the *OLED* are extracted simultaneously in the threshold voltage extraction stage, and the mobility-related discharge voltage is generated in the data input stage. The circuit not only can compensate the electrical characteristics variation, i.e., the threshold voltage variation and mobility variation, but also can compensate the *OLED* degradation. Furthermore, the circuit can prevent the *OLED* flicker, and can achieve the wide data voltage range. The circuit simulation results show that the *OLED* current error rates (CERs) are lower than 3.89% when the transistor’s threshold voltage variation is ±0.5V, lower than 3.49% when the mobility variation is ±30%.

## 1. Introduction

**Figure 1:**
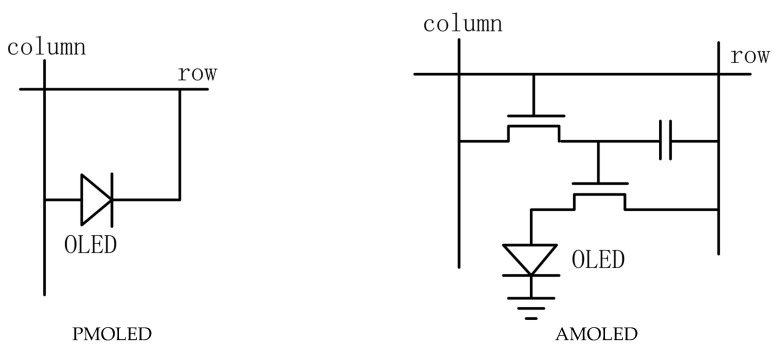
Schematic of PMOLED and AMOLED [4].

*OLED*s (organic light-emitting diodes) have gained widespread attention for their advantages such as low power consumption, high contrast, fast response time, thinner, and more foldable characteristics. According to the different driving methods, *OLED* driving technology can be divided into PMOLEDs (passive-matrix *OLED*s) and AMOLED (active-matrix *OLED*s). PMOLED has the advantages of a simple structure and low cost. However, PMOLED requires a larger driving voltage, and its power consumption is significantly higher than the AMOLED. As shown in Figure 1, AMOLEDs use independent thin-film transistors to control each pixel, so that each pixel can be continuously and independently driven and lit. Therefore, AMOLED is suitable for large and high-resolution displays, and has high application prospects for the displays [1,2,3,4].

In AMOLED pixel circuits, the oxide thin-film transistor (TFT) has great advantages, such as high carrier mobility, high light transmittance, good uniformity, and low off current, so it is widely applied to drive *OLED*, especially for large size AMOLED [5,6,7,8,9].

On the one hand, the TFT’s electrical characteristics variation, i.e., the threshold voltage variation and mobility variation would lead to the *OLED* current change. On the other hand, the *OLED* degradation would also lead to the *OLED* current change. The *OLED* current change brings out the uniformity of displays [10,11]. Therefore, in AMOLED displays, the circuit is needed to compensate the TFT’s electrical characteristics’ variation and the *OLED* degradation. Generally, the compensation circuit is divided into the current compensation circuit and the voltage compensation circuit.

The current compensation circuit can successfully compensate the TFT’s electrical characteristics’ variation [12,13]. However, the compensation speed is relatively slow at low gray level. This problem could be solved by the voltage compensation circuit [14,15,16].

Therefore, lots of valuable voltage compensation pixel circuits have been reported [17,18,19,20,21,22,23,24,25,26,27,28,29,30].

In [17,18,19,20,21], the circuits can compensate the threshold voltage variation successfully. In [22,23,24,25], the circuits can compensate both threshold voltage and mobility variations successfully. In [26,27,28], the circuits can compensate both threshold voltage variation and *OLED* degradation successfully. To obtain higher uniformity of displays, it would be better if the circuits can compensate the above three items.

In [29], the circuit can compensate the above three items successfully, but the circuit cannot prevent the *OLED* flicker. In [30], the circuit not only can compensate the above three items, but also can prevent the *OLED* flicker successfully. However, the data voltage must be less than the *OLED* threshold voltage. Thus, the data voltage range is limited.

In this paper, a voltage compensation pixel circuit is proposed. In the threshold voltage extraction stage, the circuit can extract the threshold voltages of both the TFT and the *OLED* simultaneously. In the data input stage, the circuit can generate the mobility-related discharge voltage. The simulation results show that the circuit can compensate three items: the threshold voltage variation, the mobility variation, and the *OLED* degradation, can prevent the image flicker, and can achieve the wide data voltage range.

## 2. Materials and Methods

**Figure 2:**
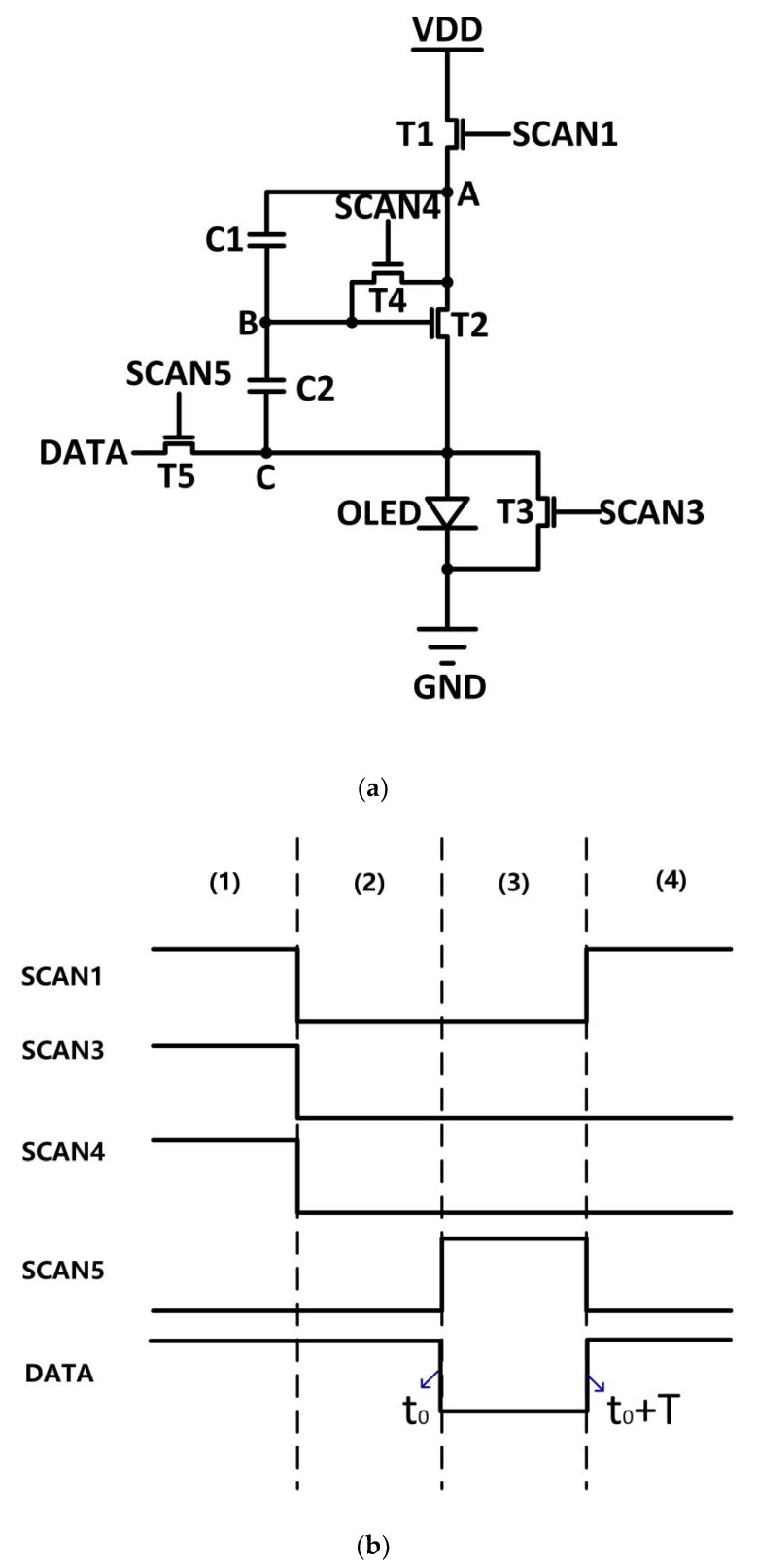
(**a**) Schematic of the proposed pixel circuit and (**b**) timing diagram: (1) initialization stage, (2) threshold voltage extraction stage, (3) data input stage, and (4) emission stage.

The circuit structure and the driving schematic diagram are shown in Figure 2. As shown in Figure 2a, the circuit consists of one driving TFT (*T*2), four switching TFTs (*T*1, *T*3, *T*4, *T*5), and two capacitors (C1, C2).

As shown in Figure 2b, the driving schematic diagram contains four stages: (1) the initialization stage, (2) the threshold voltage extraction stage, (3) the data input stage, and (4) the emission stage.

The working principle of the circuit is described as follows.

### 2.1. Initialization Stage

**Figure 3:**
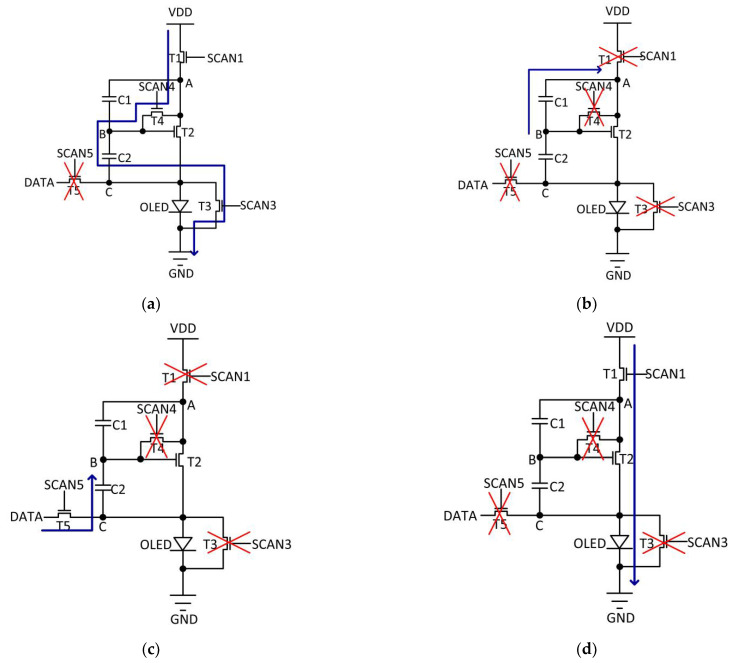
Schematic of the circuit operation in (**a**) initialization stage, (**b**) threshold voltage extraction stage, (**c**) data input stage, and (**d**) emission stage.

In the initialization stage, as shown in Figure 2b, SCAN1, SCAN3, and SCAN4 are high. SCAN5 is low. Therefore, *T*1, *T*3, and *T*4 are turned on. *T*5 is turned off. The schematic of the circuit in this stage is shown in Figure 3a.

Because *T*1 and *T*4 are turned on, the voltage of node B is charged to VDD.

Because *T*3 is turned on, no current flows through the *OLED*. Therefore, the *OLED* flicker is prevented.

### 2.2. Threshold Voltage Extraction Stage

In the threshold voltage extraction stage, as shown in Figure 2b, SCAN1, SCAN3, and SCAN4 go to low. SCAN5 remains low. Therefore, *T*1, *T*3, *T*4, and *T*5 are turned off. The schematic of the circuit in this stage is shown in Figure 3b.

Because *T*1 is turned off, no current flows through the *OLED*. Therefore, the *OLED* flicker is prevented.

The voltage of node *B* is gradually discharged until *T*2 is turned off. The voltage of node *B* goes to

(1) $$V_{B}=V_{\mathrm{TH}\_T2}+V_{\mathrm{TH}\_OLED}.$$

Consequently, the threshold voltages of both *T*2 and *OLED* are extracted simultaneously in this stage.

### 2.3. Data Input Stage

In the data input stage, as shown in Figure 2b, SCAN1, SCAN3 and SCAN4 remain low; SCAN5 goes to high. Therefore, *T*1, *T*3, and *T*4 are turned off; *T*5 is turned on. The schematic of the circuit in this stage is shown in Figure 3c.

Because *T*1 is turned off, no current flows through the *OLED*. Therefore, the *OLED* flicker is prevented.

In [29], to prevent *OLED* flicker, the data voltage range is limited: it is much less than the *OLED* threshold voltage. In this paper, the above limitation is avoided.

At the beginning and the end of this stage, *t* is defined as *t*_0_ and *t*_0_ + *T*, respectively. They are indicated in Figure 2b.

At the time *t*_0_, the data voltage (*V_DATA_*) is input to the circuit; *V_C_* and *V_B_* are expressed as

(2) $$V_{C}\left(t=t_{0}\right)=V_{DATA}+V_{TH\_OLED},$$

(3) $$V_{B}\left(t=t_{0}\right)=V_{TH_{T2}}+V_{TH\_OLED}.$$

After the time *T*, *V_C_* remains unchanged; *V_B_* discharges through *C*1, *C*2, and *T*2. At the time *t*_0_ + *T*, *V_C_* and *V_B_* are expressed as

(4) $$V_{C}\left(t=t_{0}\right)=V_{DATA}+V_{TH\_OLED},$$

(5) $$V_{B}\left(t=t_{0}\right)=V_{TH\_T2}+V_{TH\_OLED}-\left(V_{DATA}+V_{TH\_OLED}\right)\times \frac{C2}{C1+C2}-\Delta V\mu .$$

where ΔVμ is the discharged voltage related to the mobility of *T*2.

The expression of ΔVμ is derived as follows. When *DATA* is input to the circuit, *T*2 keeps the diode-connected structure. Therefore, the mobility-related discharge voltage ΔVμ is stored in *C*1. By the law of discharge conservation, we have [30]

(6) $$\left(C1+C2\right)\frac{dV_{GS\_T2}}{dt}=\frac{1}{2}\mu C_{OX}\frac{W}{L}{\left(V_{GS\_T2}-V_{TH\_T2}\right)}^{2}.$$

where μ is the mobility of *T*2, C_OX_ is the gate oxide capacitance per unit area, and $\frac{W}{L}$ is the width–length ratio of *T*2.

Integrating (6), we have

(7) $$\int_{V_{GS\_T2}\left(t=t_{0}\right)}^{V_{GS\_T2}\left(t=t_{0}+T\right)}\frac{1}{{\left(V_{GS\_T2}-V_{TH\_T2}\right)}^{2}}dV_{GS\_T2}=\int_{t=t_{0}}^{t=t_{0}+T}\frac{\mu C_{OX}\frac{W}{L}}{2\left(C_{1}+C_{2}\right)}dt.$$

where

(8) $$\;V_{GS\_T2}\left(t=t_{0}\right)=V_{B}\left(t=t_{0}\right)-V_{C}\left(t=t_{0}\right),$$

(9) $$V_{GS\_T2}\left(t=t_{0}+T\right)=V_{B}\left(t=t_{0}+T\right)-V_{C}\left(t=t_{0}+T\right).$$

Substituting (2)–(5) to (9), we obtain

(10) $$\Delta V\mu =\left(V_{DATA}+V_{TH\_OLED}\right)\times \frac{C2}{C1+C2}-\frac{1}{\frac{\mu C_{OX}\frac{W}{L}}{2\left(C_{1}+C_{1}\right)}T+\frac{1}{\left(V_{DATA}+V_{TH\_OLED}\right)\times \frac{C2}{C1+C2}}}.$$

Consequently, the mobility-related discharge voltage ΔVμ is generated in this stage.

### 2.4. Emission Stage

In the emission stage, as shown in Figure 2b, SCAN1 goes to high, SCAN3 and SCAN4 remain low, and SCAN5 goes to low. Therefore, *T*1 is turned on, and *T*3, *T*4, and *T*5 are turned off.

The schematic of the circuit in this stage is shown in Figure 3d.

The driving TFT (*T*2) operates in the saturation region; the *OLED* current is expressed as follows:

(11) $$I_{OLED}=\frac{1}{2}{\mu C}_{\mathrm{OX}}\frac{W}{L}{\left(V_{\mathrm{GS}\_T2}-V_{\mathrm{TH}\_T2}\right)}^{2}.$$

Substituting (9) to (11), we obtain

(12) $$I_{OLED}=\frac{1}{2}\mu C_{OX}\frac{W}{L}{\left(\left(V_{DATA}+V_{TH\_OLED}\right)\times \frac{C2}{C1+C2}-V_{DATA}-\Delta V\mu \right)}^{2}.\;$$

Substituting (10) to (12), we obtain

(13) $$I_{OLED}=\frac{1}{2}\mu C_{OX}\frac{W}{L}{\left(\frac{1}{\frac{\mu C_{OX}\frac{W}{L}}{2\left(C_{1}+C_{1}\right)}T+\frac{1}{\left(V_{DATA}+V_{TH\_OLED}\right)\times \frac{C2}{C1+C2}}}-V_{DATA}\right)}^{2}.$$

From (12), it is found that the *OLED* current is independent of the threshold voltage *V_TH_*__*T*2_. That is, when *V_TH_*__*T*2_ varies, *I_OLED_* remains stable. Therefore, the circuit can compensate the threshold voltage variation.

From (10), it is found that when the mobility μ increases, ΔVμ will increase, and vice versa. Consequently, in (12), when the mobility varies, *I_OLED_* remains stable. Therefore, the circuit can compensate the mobility variation. This point also can be explained by (13). In (13), when μ increases, both $\frac{1}{2}\mu C_{OX}\frac{W}{L}$ and $\frac{\mu C_{OX}\frac{W}{L}}{2\left(C_{1}+C_{1}\right)}T$ will increase; thus, *I_OLED_* remains stable, and vice versa.

From (13), it is found that the *OLED* current is positively correlated with *V_TH_*__*OLED*_. Therefore, the circuit can compensate the *OLED* degradation [29,30,31].

## 3. Results and Discussions

**Figure 4:**
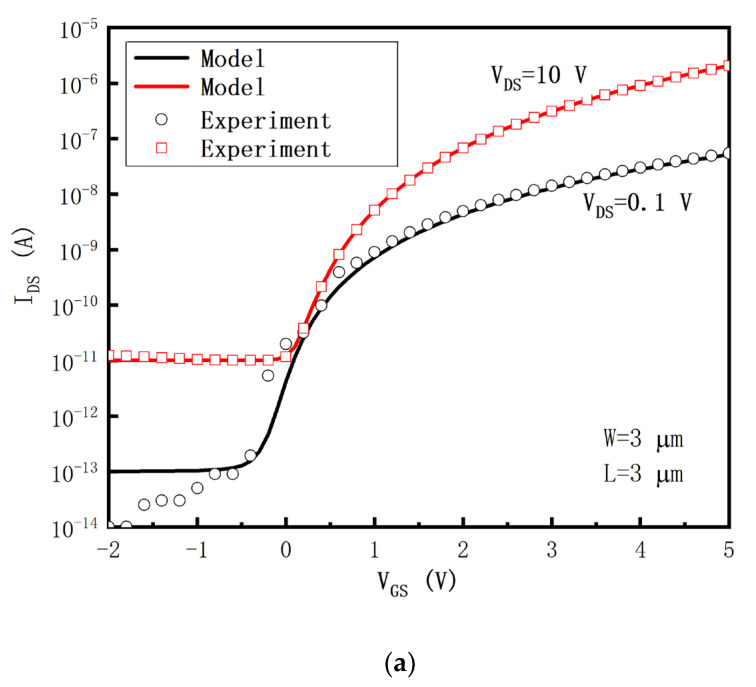

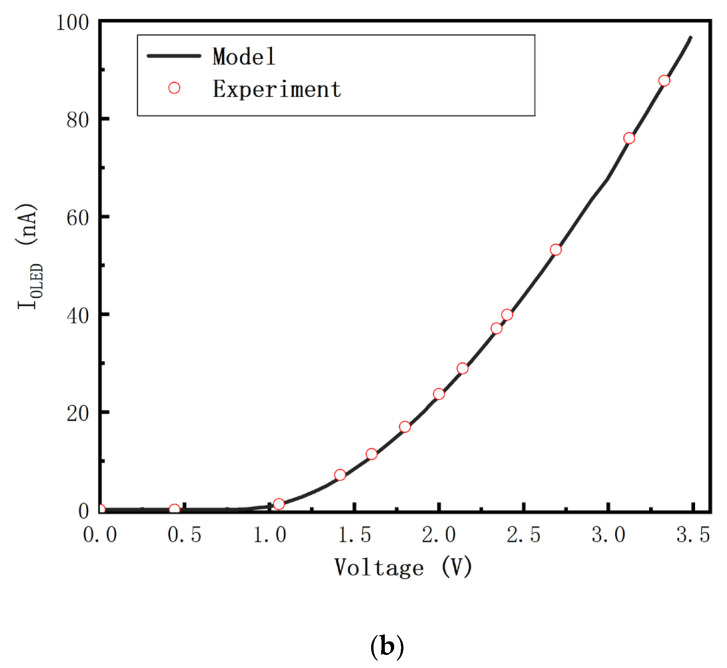
(**a**) The transfer characteristic of the a-IGZO TFT [8], and (**b**) the electrical characteristic of the *OLED* [33].

In the circuit simulation, to evaluate the compensation performance, the SPICE model (level = 35) is used for the oxide TFTs. The TFTs’ threshold voltage and mobility are 1.5 V and 50 cm^2^/V, respectively. The TFTs’ threshold voltage variation and mobility variation are ±0.5 V and ±30%, respectively [9,14,28]. The *OLED* model is equivalent to a TFT and a *C_OLED_* in parallel [22,27,32]. The oxide TFT and the *OLED* models are verified by the experimental data [8,33], which are shown in Figure 4.

The values of the design parameters are shown in Table 1; the range of the values is reasonable, which is consistent with the previous pixel circuit applications [11,19,20,27,32,34].

**Figure 5:**
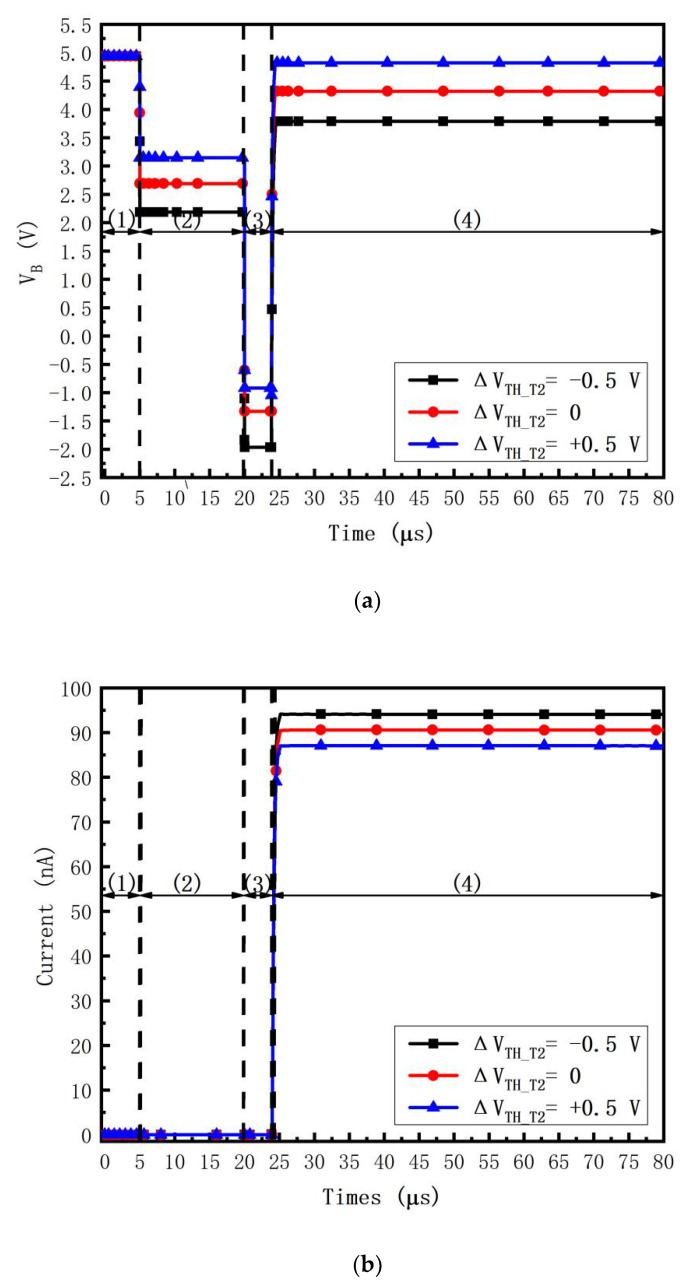

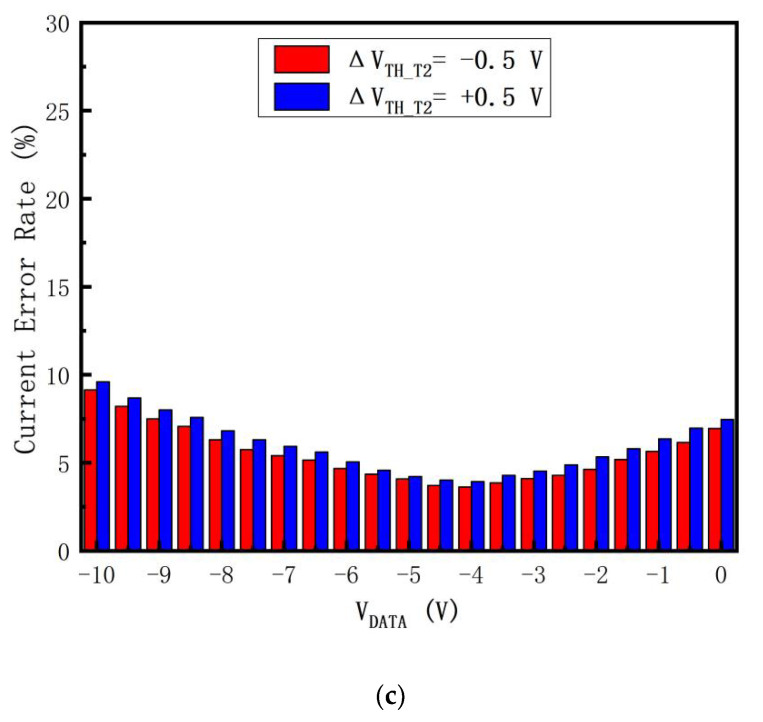
Transient waveforms of (**a**) *V_B_* and (**b**) *I_OLED_* when Δ*V*_*TH*_*T*2_ = −0.5, 0, and +0.5 V at *V_DATA_* = −4 V, where (1) initialization stage, (2) threshold voltage extraction stage, (3) data input stage, (4) emission stage, and (**c**) current error rates versus *V_DATA_* when the threshold voltage varies.

Figure 5a shows the transient waveforms of *V_B_*, i.e., the gate voltage of *T*2, at *V_DATA_* = −4 V. It is found that when Δ*V*_*TH*_*T*2_ = ±0.5 V, Δ*V_B_* approximates ±0.5 V in the threshold voltage extraction stage, i.e., *V_B_* senses the threshold voltage variation successfully. Figure 5b shows the transient waveforms of the *OLED* current *I_OLED_*. It is found that *I_OLED_* = 0 except for the emission stage, i.e., the *OLED* flicker is prevented. In the emission stage, when Δ*V*_*TH*_*T*2_ = −0.5, 0, and +0.5 V, the transient waveforms of *I_OLED_* = 94.11, 90.59, and 87.06 n A, respectively. The current error rates (CERs) are 3.74% and 0, 3.89%, respectively. Thus, the circuit compensates Δ*V*_*TH*_*T*2_ successfully.

The CER for Δ*V*_*TH*_*T*2_ is defined as

(14) $$\frac{I_{OLED}\left({\Delta V}_{\mathrm{TH}\_T2}=0\right)-I_{OLED}\left({\Delta V}_{\mathrm{TH}\_T2}=\pm 0.5\;V\right)}{I_{OLED}\left({\Delta V}_{\mathrm{TH}\_T2}=0\right)}\times 100\%.$$

Figure 5c shows the transient waveforms of CER vary when Δ*V*_*TH*_*T*2_ is +0.5 V and −0.5 V, respectively. It is found that the CERs are less than ±9.59% within the whole data range. Thus, the threshold voltage variation Δ*V*_*TH*_*T*2_ is compensated successfully.

**Figure 6:**
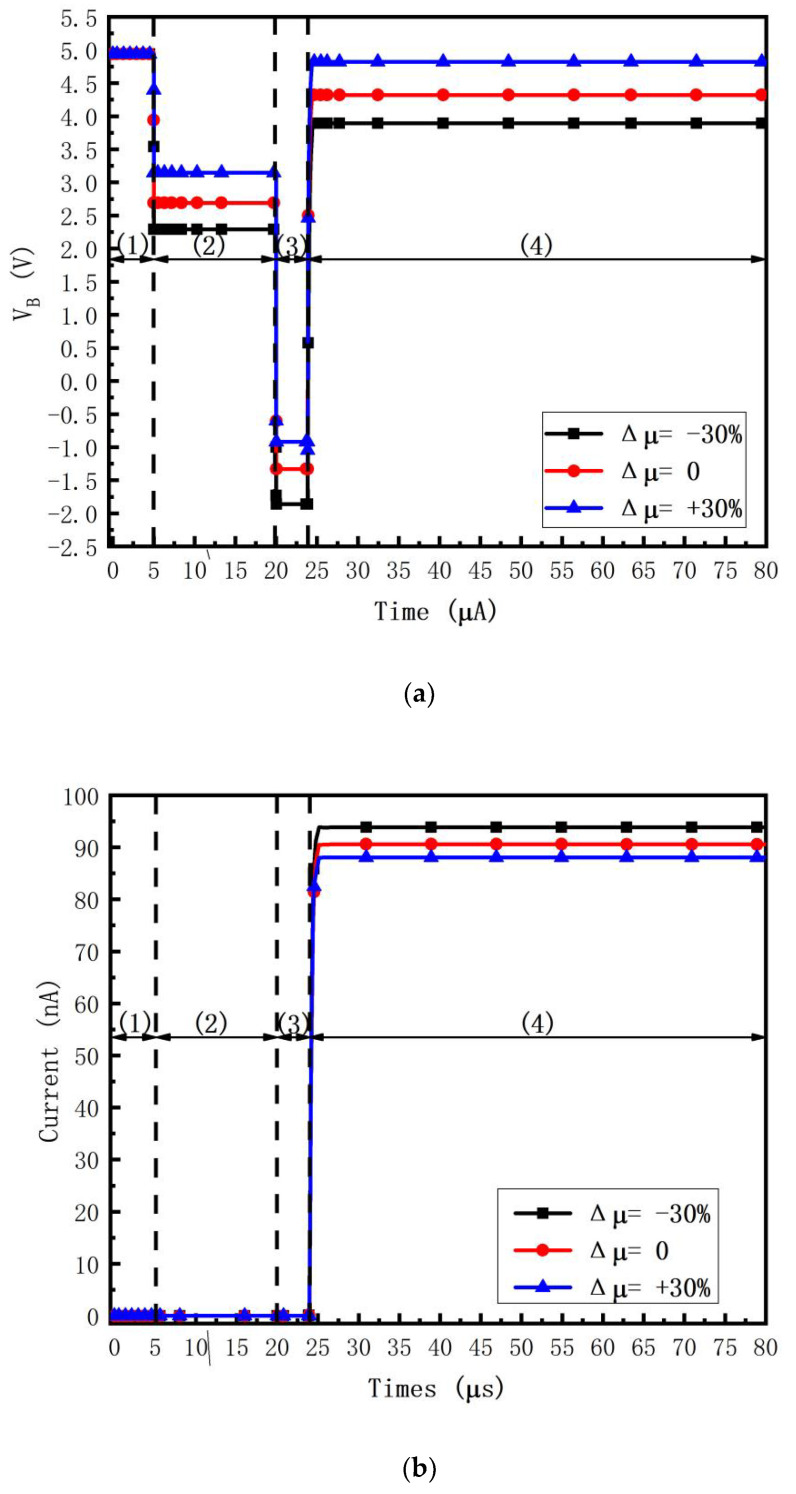

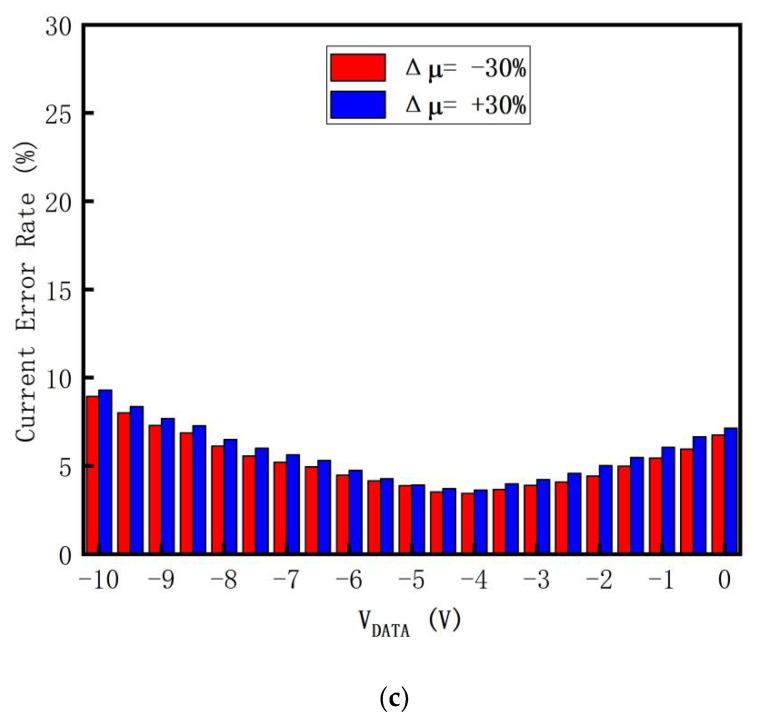
Transient waveforms of (**a**) *V_B_* and (**b**) *I_OLED_* when Δu = +30%, 0, and −30% at *V_DATA_*= −4V, where (1) initialization stage, (2) threshold voltage extraction stage, (3) data input stage, (4) emission stage, and (**c**) current error rates versus *V_DATA_* when the threshold voltage varies.

Figure 6a shows the transient waveforms of *V_B_* at *V_DATA_* = −4V. It is found that when Δu= ±30%, the variation of *V_B_* is similar to Δu, i.e., *V_B_* senses the mobility variation successfully. Figure 6b shows the transient waveforms of the *OLED* current *I_OLED_*. It is found that *I_OLED_* = 0 except for the emission stage, i.e., the *OLED* flicker is prevented. In the emission stage, when Δu = −30, 0, and +30%, the transient waveforms of *I_OLED_* = 88.06, 90.59, and 93.87 n A, respectively. The current error rates are 2.81% and 0, 3.49%, respectively. Thus, the circuit compensates Δu successfully.

The CER for Δu is defined as

(15) $$\frac{I_{OLED}\left(\Delta u=0\right)-I_{OLED}\left(\Delta u=\pm 30\%\right)}{I_{OLED}\left(\;\Delta u=0\right)}\times 100\%.$$

Figure 6c shows the transient waveforms of CER varies when Δu is +30% and −30%, respectively. It is found that the CERs are less than ±9.28% within the whole data range. Thus, the mobility variation Δu is compensated successfully.

In this paper, the circuit can compensate the *OLED* degradation. It is explained as follows. For the long time operation, the *OLED* luminance degrades while V_TH_*OLED*_ increases [29,30,31]. Therefore, *I_OLED_* (13) increases. The increase in *I_OLED_* brings about the increase in the *OLED* luminance. Thus, the *OLED* luminance degradation is compensated.

In Figure 5a,b and Figure 6a,b, the time of the third stage, i.e., the data input stage, is set to 3.8 us. It is suitable for the 8K4K ultrahigh definition (7680 × 4320, UHD) for high-performance display [11,35,36].

As shown in Table 2, the valuable publications are compared with this paper. In [29], the circuit can compensate the threshold voltage variation, the mobility variation, and the *OLED* degradation successfully, but the circuit cannot prevent the *OLED* flicker. In [30] and this paper, the circuits not only can compensate the above three items successfully, but also can prevent the *OLED* flicker.

However, in [30], *V_DATA_* must be less than V_TH_*OLED*_, that is, the range of *V_DATA_* is limited. In this paper, the range of *V_DATA_*_is_ not limited by V_TH_*OLED*_. Therefore, the circuit achieves the wide data voltage range.

**Figure 7:**
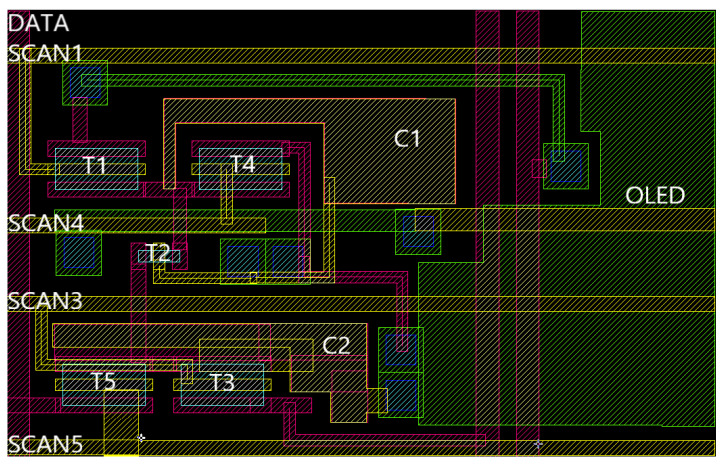
Layout structure of the circuit.

Figure 7 shows the layout structure of the circuit. SCAN1, SCAN3, SCAN4, and SCAN5 are transverse lines, which are set to 4 um. These transverse lines are shared by the pixels of the same row. VDD, GND, and DATA are the vertical lines, which are set to 6 um. VDD and GND are shared by the entire display panel. DATA is shared by the pixels of the same column. The total layout area is 180 um × 110 um. The proposed layout achieves an aperture ratio of 39.14%.

## 4. Conclusions

The pixel circuit is presented for improving AMOLED displays uniformity. In the threshold voltage extraction stage, the threshold voltages of the driving TFT and the *OLED* are extracted. In the data input stage, the discharge voltage related to mobility is generated. Consequently, the circuit not only compensates the threshold voltage variation, the mobility variation, and the *OLED* degradation, but also prevents the image flicker and achieves the wide data voltage range.

## Figures and Tables

**Table 1:** Design parameters of the circuit.

Parameters (Unit)	Value	Parameters (Unit)	Value
*W*_*T*2_ (µm)	3	VDD (V)	5
*L*_*T*2_ (µm)	3	*C*2 (pF)	0.2
*W*_*T*1,*T*3,*T*4,*T*5_ (µm)	22	*C_OLED_* (pF)	0.2
*L*_*T*1,*T*3,*T*4,*T*5_ (µm)	3	SCAN_1,3,4,5_ (V)	−5~5
*C*1 (pF)	0.4	*DATA* (V)	*V_DATA_*~0

**Table 2:** Comparison between this paper and the previous publications.

Publications	Structure	*V_TH_* Compensation	*µ* Compensation	*OLED* Degradation	Prevent Image Flicker
2015 [22]	5*T*2C	√	√	-	√
2015 [27]	5*T*2C	√	-	√	√
2015 [28]	4*T*1C	√	-	√	√
2016 [30]	4*T*2C	√	√	√	√
2017 [26]	4*T*1C	√	-	√	√
2018 [23]	6*T*2C	√	√	-	√
2018 [29]	5*T*2C	√	√	√	-
2020 [19]	6*T*1C	√	-	-	√
2020 [20]	9*T*2C	√	-	-	√
2022 [17]	6*T*2C	√	-	-	√
This paper	5*T*2C	√	√	√	√

## Data Availability

Data sharing is not applicable to this article as no new data were created or analyzed in this study.
